# Effects of Chitin and Its Derivative Chitosan on Postharvest Decay of Fruits: A Review

**DOI:** 10.3390/ijms12020917

**Published:** 2011-01-27

**Authors:** Hongyin Zhang, Renping Li, Weimin Liu

**Affiliations:** College of Food and Biological Engineering, Jiangsu University, Zhenjiang 212013, Jiangsu, China; E-Mails: renpingli1121@163.com (R.L.); liuwmwu@ujs.edu.cn (W.L.)

**Keywords:** chitin, chitosan, fruits, postharvest decay, control efficacy, mechanisms

## Abstract

Considerable economic losses to harvested fruits are caused by postharvest fungal decay during transportation and storage, which can be significantly controlled by synthetic fungicides. However, considering public concern over pesticide residues in food and the environment, there is a need for safer alternatives for the control of postharvest decay to substitute synthetic fungicides. As the second most abundant biopolymer renewable source in nature, chitin and its derivative chitosan are widely used in controlling postharvest decay of fruits. This review aims to introduce the effect of chitin and chitosan on postharvest decay in fruits and the possible modes of action involved. We found most of the actions discussed in these researches rest on physiological mechanisms. All of the mechanisms are summarized to lay the groundwork for further studies which should focus on the molecular mechanisms of chitin and chitosan in controlling postharvest decay of fruits.

## 1. Introduction

Fresh fruits and vegetables are very perishable and susceptible to postharvest diseases which limit the storage period and marketing life of them. Moreover, postharvest decay results in substantial economic losses around the world. As is known, synthetic fungicide treatment has long been the main method for controlling postharvest diseases [1]. However, there is increasing international concern over the indiscriminate use of synthetic fungicides on crops because of the possible harmful effects on human health [2] and the emergence of pathogen resistance to fungicides [3]. Therefore, new alternatives for controlling postharvest diseases which have good efficacy, low residues, and little or no toxicity to non-target organisms are in urgent demand.

A great many alternative strategies, including biological control with antagonistic microorganisms, applications of plant bioactive compounds, refrigerated storage, heat treatment, high pressure processing and modified atmosphere storage [4,5], have been used to control postharvest diseases of fruits and inhibit growth of the pathogens. However, all these methods could not control postharvest diseases as effectively as synthetic fungicides. And some of the processes also have limitations, such as partial destruction of quality attributes of food products, especially heat-labile nutrients and sensory attributes [6].

Chitin, together with its derivative chitosan, has been reported as a promising alternative to control postharvest diseases. Chitin is the second most abundant biopolymer renewable source in nature after cellulose, which has a versatile application potential in the agriculture-food industry [7], for instance, as a biopesticide, which has been approved by the U.S. Environmental Protection Agency (EPA), and as a food additive, approved by the U.S. Food and Drug Administration (FDA). Similarly, chitosan has become a prospective alternative treatment for fruit and vegetables due to its natural character, antimicrobial activity, and elicitation of defense responses in plant tissue [8,9]. Chitin or chitosan has been used to control postharvest diseases of many fruits such as pear [10], strawberry [11,12], table grape [13], tomato [14], citrus [15], and longan [16].

This review summarizes the efficiency of chitin and chitosan on controlling postharvest diseases which consequently prolongs the shelf-life of fruits, and its possible mechanisms involved. New research approaches to fully understand the mechanism of chitin and chitosan against fungal pathogens are also suggested.

## 2. Structures and Functions of Chitin and Chitosan

**Figure 1.**
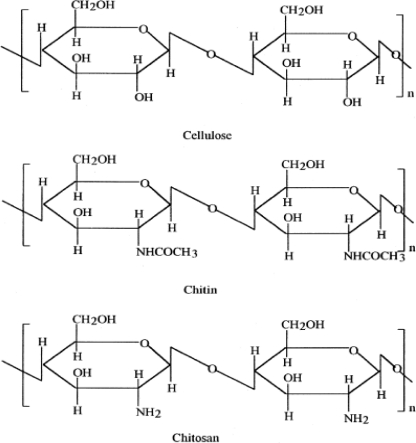
Structures of cellulose, chitin and chitosan. Reproduced from Reference [20].

Chitin and chitosan are polysaccharides, chemically similar to cellulose, differing only by the presence or absence of nitrogen [17]. Chitin, a naturally abundant mucopolysaccharide, and the supporting material of crustaceans, insects, *etc*., is well known to consist of 2-acetamido-2-deoxy-β-d-glucose though a β (1→4) linkage. Chitin can be degraded by chitinase. Its immunogenicity is exceptionally low, in spite of the presence of nitrogen. It is a highly insoluble material resembling cellulose in its solubility and low chemical reactivity. It may be regarded as cellulose with hydroxyl at position C-2 replaced by an acetamido group. Like cellulose, it functions naturally as a structural polysaccharide. Chitin is a white, hard, inelastic, nitrogenous polysaccharide and the major source of surface pollution in coastal areas. Chitosan is the *N*-deacetylated derivative of chitin, although this *N*-deacetylation is almost never complete. A sharp nomenclature with respect to the degree of *N*-deacetylation has not been defined between chitin and chitosan [18,19]. The structures of cellulose, chitin and chitosan are shown in Figure 1 [20].

Chitin and its derivative chitosan have been of interest in the past few decades due to their potential broad range of industrial applications [21,22]. However, there has been limited attention paid to the food application of these versatile biopolymers. They offer a wide range of unique applications including bioconversion for the production of value-added food products [23–25], preservation of foods from microbial deterioration [26–30], formation of biodegradable films [31–36], recovery of waste material from food processing discards [37–44], purification of water [45–48] and clarification and deacidification of fruit juices [49–53]. In this text, we especially pay attention to the antimicrobial function of chitin and chitosan in fruits. Hernández-Lauzardo *et al.* have reported the significant effect of chitosan on inhibiting three isolates of *Rhizopus stolonifer* obtained from several fruits [54]. It has been reported that chitin and chitosan are effective in reducing postharvest diseases of fruits and vegetables by inhibiting spore germination, germ tube elongation, mycelial growth of fungal phytopathogens, enhancing the efficacy of antagonistic yeasts, and boosting the activity of defense-related enzymes or pertinent substances [55–60].

## 3. Effects of Chitin and Chitosan on Postharvest Disease of Fruits

**Figure 2.**
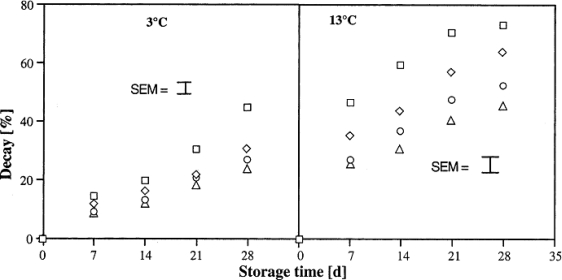
Effect of pre-harvest chitosan spray treatments on the decay of strawberry fruit stored at 3 (S.E.M. ± 1.58) and 13 °C (S.E.M. ± 2.28). Control (□); 2 gl^−1^ (⋄); 4 gl^−1^ (○) and 6 gl^−1^ (Δ). Data were pooled across the number of sprays and picks and repetitions. Reproduced from Reference [12].

**Figure 3.**
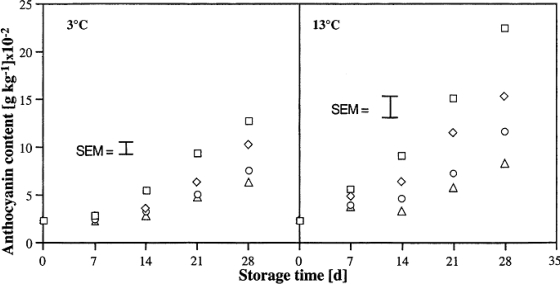
Effect of pre-harvest chitosan spray treatments on anthocyanin content of strawberry fruit stored at 3 (S.E.M. ± 0.57 × 10^−2^) and 13 °C (S.E.M. ± 0.84 × 10^−2^). Control (□); 2 gl^−1^ (⋄); 4 gl^−1^(○) and 6 gl^−1^ (Δ). Data were pooled across the number of sprays and picks and repetitions. Reproduced from Reference [12].

**Figure 4.**
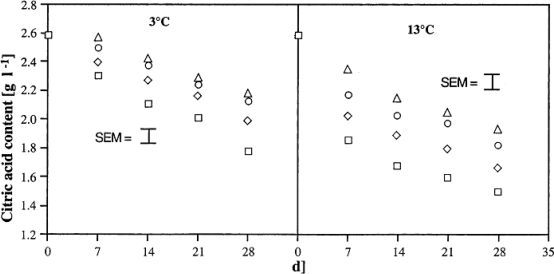
Effect of pre-harvest chitosan spray treatments on titratable acidity of strawberry fruit stored at 3 (S.E.M. ± 0.038) and 13 °C (S.E.M. ± 0.18); Control (□); 2 gl^−1^ (⋄); 4 gl^−1^(○) and 6 gl^−1^ (Δ). Data were pooled across the number of sprays and picks and repetitions. Reproduced from Reference [12].

Recently, the method of using chitin and chitosan to control postharvest diseases of fruits was developed. Chitosan at low molecular weight (LMWC) has been reported to control postharvest diseases of citrus fruit [15]. The results indicated that LMWC significantly inhibited the decay of citrus fruit caused by *Penicillium digitatum*, *Penicillium italicum*, *Botrydiplodia lecanidion*, and *Botrytis cinerea* after 14 days storage at 25 °C, and is more effective than TBZ and high molecular weight chitosan (HMWC) (Table 1). Meanwhile, low molecular weight chitosan coating beneficially influenced firmness, total soluble solid content, titratable acidity, ascorbic acid content and water content of citrus fruit after 56 days of storage at 15 °C (Table 2). Bhaskara Reddy *et al*. [12] found that pre-harvest chitosan sprays effectively inhibited the postharvest decay of strawberry fruit caused by *Botrytis cinerea* during storage at 3 and 13 °C, and the decay decreased with increasing chitosan concentration (Figure 2). Furthermore, fruits from chitosan sprayed plants were firmer and ripened at a slower rate as indicated by anthocyanin content and titratable acidity than berries from non-treated plants (Figures 3 and 4).

In addition to being singularly applied, there are many reports on combined applications of chitosan with other antifungal compounds. Yu *et al*. [61] found that chitosan applied alone or with *Cryptococcus laurentii* could effectively inhibit the blue mold rot caused by *Penicillium expansum* in apple fruit after seven days of incubation at 20 °C. When applied alone, treatment with chitosan at the highest concentration (1%) and the lowest viscosity (12 cP) was the most effective. When used in combinations, treatment of *Cryptococcus laurentii* with chitosan at a concentration of 0.1% and lowest viscosity (12 cP) was the most effective (Table 3). Similar results were found when using chitosan coating with postharvest calcium on extending shelf-life of strawberries [62]; and chitosan with ethanol on controlling postharvest gray mold of table grapes caused by *Botrytis cinerea* [63].

**Figure 5.**
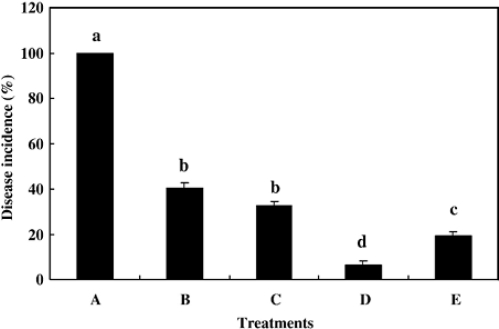
Efficacy of *Cryptococcus laurentii* (1 × 10^8^ cells/mL) in inhibiting disease incidence of blue mold caused by *Penicillium expansum* in pear fruit wounds after 6 days of incubation at 25 °C in nutrient yeast dextrose broth (NYDB) (**B**), NYDB amended with chitin at 2.0% (**C**), 1.0% (**D**), 0.5% (**E**). The treatment with water and inoculated with *P. expansum* was served as the positive control (**A**). Bars represent standard errors. Different letters indicates significant differences (*P* = 0.01) according to the Duncan’s multiple range test. Reproduced from Reference [10].

**Figure 6.**
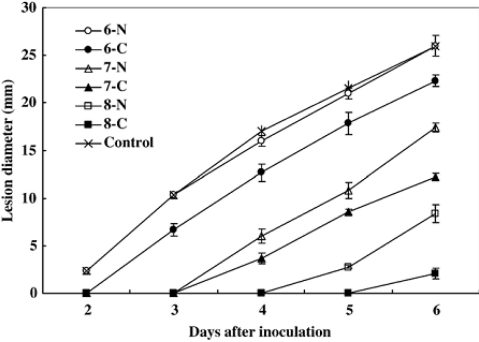
Efficacy of *Cryptococcus laurentii* in inhibiting the lesion diameter of blue mold caused by *Penicillium expansum* in pear fruit wounds at 25 °C. Treatment with water and inoculated with *P. expansum* was applied as the control. Bars represent standard errors. (○) *C. laurentii* grown in NYDB at 1 × 10^6^ cells/mL; (•) *C. laurentii* grown in NYDB with chitin at 1.0% at 1 × 10^6^ cells/mL; (Δ) *C. laurentii* grown in NYDB at 1 × 10^7^ cells/mL; (▴) *C. laurentii* grown in NYDB with chitin at 1.0% at 1 × 10^7^ cells/mL; (□) *C. laurentii* grown in NYDB at 1 × 10^8^ cells/mL; (▪) *C. laurentii* grown in NYDB with chitin at 1.0% at 1 × 10^8^ cells/mL. Reproduced from Reference [10].

**Figure 7.**
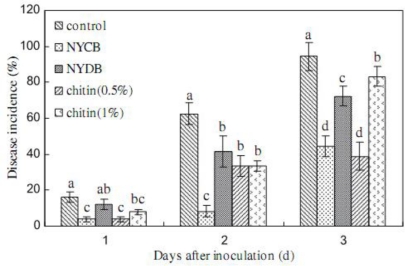
Efficacy of *R. glutinis* harvested from different media in controlling grey mould decay of strawberries. Each value is the mean of two experiments. Bars represent standard deviations. Different letters indicate significant differences (*P* = 0.05) according to Duncan’s multiple range test and the data from each time point are separated. Reproduced from Reference [11].

Biological antagonists have already been shown to effectively inhibit the postharvest decay of fruit in recent years [64–67]. However, for biological control to be accepted as an economically viable option, consistency and efficacy of antagonistic yeasts in controlling postharvest diseases must be enhanced [68,69]. Many attempts have been proposed to improve the performance of postharvest biocontrol yeasts. Physiological manipulation may be a useful method [69]. Recently, enhancement of the biocontrol efficacy of antagonists to postharvest diseases of fruits by addition of chitin or chitosan to the growth medium was reported. Yu *et al*. [10] found that the disease incidence and lesion diameter of blue mold rot caused by *Penicillium expansum* in pear fruit was significantly inhibited by *Cryptococcus laurentii* which was cultivated in nutrient yeast dextrose broth (NYDB) media amended with chitin, especially at the optimal concentration (1.0%) (Figures 5 and 6). Similarly, our research team used *Rhodotorula glutinis* cultivated with NYDB amended with chitin or nutrient yeast chitin broth (NYCB: Chitin as the sole carbon source instead of dextrose in the media of nutrient yeast dextrose broth) to control the grey mold decay caused by *Botrytis cinerea* in strawberries [11], we found that the antagonistic activity of *R. glutinis* was greatly enhanced by chitin inducing incubation (0.5% chitin), which resulted in a significant reduction of the disease incidence (Figure 7).

## 4. Mode of Action of the Control of Postharvest Decay of Fruits by Chitin and Chitosan

Because of the positive charge on the C_2_ of the glucosamine monomer below pH 6, chitosan is more soluble and has a better antimicrobial activity than chitin [70]. Therefore, the application on controlling postharvest decay of fruits and the possible mechanisms discussed mostly rest on chitosan. The exact mechanism of the antimicrobial action of chitin, chitosan, and their derivatives is still imperfectly known, but different mechanisms have been proposed [71].

### 4.1. The Direct Effect of Chitin and Chitosan on Fungal Pathogens

**Figure 8.**
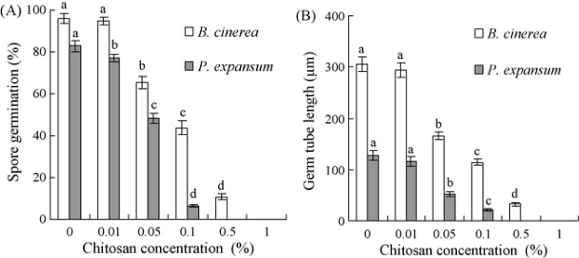
Effects of chitosan concentration on spore germination (**A**) and germ tube elongation (**B**) of *Botrytis cinerea* and *Penicillium expansum* 12 h after incubation at 25 °C. Bars represent standard deviations of the means. Values followed by different letters are significantly different according to Duncan’s multiple range test at *P* < 0.05. Reproduced from Reference [59].

**Figure 9.**
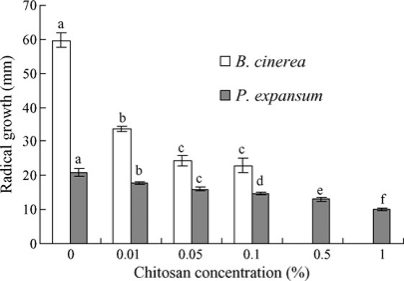
Effects of chitosan concentration on mycelial growth of *B. cinerea* and *P. expansum* 3 days after incubation at 25 °C. Bars represent standard deviations of the means. Values followed by different letters are significantly different according to Duncan’s multiple range test at *P* < 0.05. Reproduced from Reference [59].

**Figure 10.**
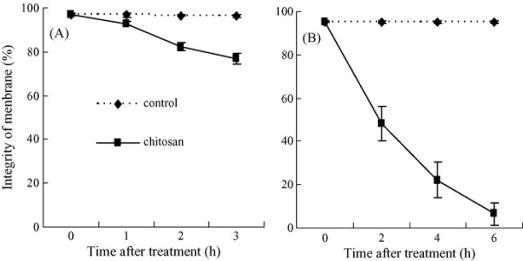
Effects of chitosan on plasma membrane integrity of the spores of *B. cinerea* (**A**) and *P. expansum* (**B**). Pathogen spores were cultured in PDB containing 5% chitosan or in PDB without chitosan as the control at 25 °C. Bars represent standard deviations of the means. Reproduced from Reference [59].

Numerous previous studies have shown that chitosan could directly inhibit spore germination, germ tube elongation and mycelial growth of many phytopathogens, such as *Botrytis cinerea* [58,59,72], *Fusarium solani* [73], *Rhizopus stolonifer* [58,74], *Penicillium* [59,72], and *Sclerotium rolfsii* [73]. Liu *et al*. [59] reported that chitosan completely inhibited spore germination of *P. expansum* at 0.5% and *B. cinerea* at 1%, significantly inhibited germ tube elongation of both pathogens when the concentration was higher than 0.01% (*P* < 0.05) (Figures 8 and 9), and the plasma membranes of spores of both pathogens were damaged (Figure 10). The reasons for the antimicrobial character of chitosan remain controversial. Two hypotheses are as follows: (1) The polycationic chitosan consumes the electronegative charges on cell surfaces and the cell permeability is changed, thus this interaction results in the leakage of intracellular electrolytes and proteinaceous constituents; (2) chitosan enters fungal cells and then essential nutrients are adsorbed, which inhibit or slow down the synthesis of mRNA and protein [75–79].

### 4.2. The Induced Disease Resistance of Fruits by Chitin and Chitosan

**Figure 11.**
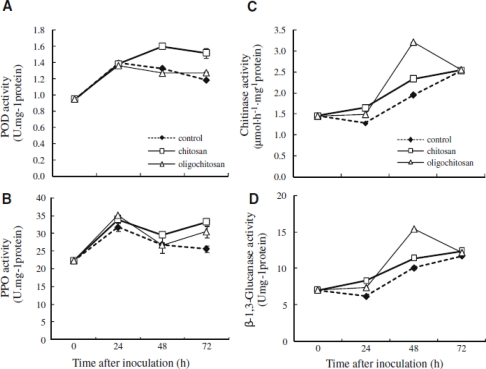
Effects of chitosan or oligochitosan on activities of POD (**A**), PPO (**B**), CHI (**C**) and β-1,3-glucanase (**D**) of pear fruit. Reproduced from Reference [87].

**Figure 12.**
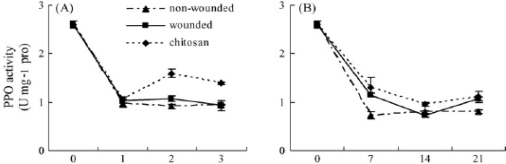

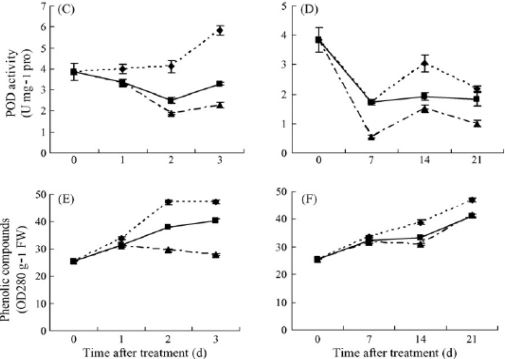
Changes of PPO activities (**A** and **B**), POD activities (**C** and **D**), and phenolic compounds (**E** and **F**) in tomato fruit. Fruit were treated with 1% chitosan, and stored at 25 °C (**A**, **C** and **E**) and 2 °C (**B**, **D** and **F**), respectively. Fruit wounded and treated with water, and non-wounded, served as controls. Bars represent standard deviations of the means. Reproduced from Reference [88].

The chitinase activity is usually induced in the presence of chitin, which may have diverse biological roles including the antifungal activity [80–82]. As an exogenous elicitor, chitosan can induce resistance in the host by increasing the activities of several defense-related enzymes, such as chitinase and β-1,3-glucanase in oranges, strawberries and raspberries [83,84], and phenylalanine ammonialyase (PAL) activity in strawberries and table grapes [85,86]. Similar results were also found by Meng *et al*. [87] in pear fruit. After being treated with chitosan or oligochitosan, the activities of POD, PPO, CHI, and β-1,3-GLU in pear fruit were induced which might be beneficial to fruit against infection by fungal pathogens (Figure 11). Liu *et al*. [88] found that treatment of chitosan induced the activities of PPO and POD, and increased the content of phenolic compounds in tomato fruit stored at 25 and 2 °C (Figure 12); these results possibly being related to the effective control of chitosan on gray mold rot and blue mold rot of tomato fruit. Moreover, chitosan is known to elicit plant defense responses by activating pathogenesis-related (PR) gene functions, such as chitinases [89,90], chitosanase, β-glucanases and lignin [91] and callose [92].

## 5. Conclusions

In conlusion, chitin and its derivative chitosan have shown a great potential as natural biodegradable substances which have anti-microbial activities. Previous studies have indicated that chitin and chitosan could effectively inhibit postharvest diseases of fruits by direct inhibition on spore germination, germ tube elongation and mycelial growth of phytopathogens and indirect inducement of defense-related enzymes, such as POD, PPO, PAL, GLU. However, the mode of action for chitin and chitosan controlling postharvest diseases of fruits are still limited and unclear. Therefore, to fully understand the mechanism of chitin and chitosan against fungal pathogens and the function in inducing defense response of fruits to pathogen infection, new approaches at the molecular and proteomic level, including the separation and identification of differential expression genes and differential expression proteins, are really needed in further studies.

## Figures and Tables

**Table 1.** Effect of low molecular weight chitosan (LMWC) on decay of citrus fruits caused by *Penicillium digitatum*, *Penicillium italicum*, *Botrydiplodia lecanidion* and *Botrytis cinerea*. Reproduced from Reference [15].

**Treatments^1^**	**Infected Fruit (%)^2^,^3^**			
	***P. digitatum***	***P. Italicum***	***B. Lecanidion***	***B. cinerea***
Control	100 ^a^	100 ^a^	100 ^a^	100 ^a^

		HMWC		
0.05%	60.0 ^c^	65.0 ^c^	78.3 ^c^	70.0 ^c^
0.1%	53.3 ^d^	66.7 ^c^	75.0 ^c^	68.3 ^c^
0.2%	41.7 ^e^	58.3 ^d^	73.3 ^c^	60.0 ^d^

		LMWC		
0.05%	21.7 ^f^	25.0 ^e^	33.3 ^d^	30.0 ^e^
0.1%	10.0 ^g^	15.3 ^f^	21.6 ^e^	18.3 ^f^
0.2%	5.0 ^h^	8.3 ^g^	15.0 ^f^	11.7 ^g^

		TBZ		
0.1%	78.3 ^b^	83.3 ^b^	88.3 ^b^	85.0 ^b^

1 The *P. digitatum*, *P. italicum*, *B. lecanidion* and *B. cinerea* spore concentrations were 10^5^ conidia/mL;

2 Means are averaged over three trials. Each trial involved treating three identical groups of 120 citrus fruit with each treatment. Decay was evaluated after 14 days of storage at 25 °C;

3 Values followed by the same letter do not significantly differ at *P* > 0.05, according to Duncan’s multiple range test.

**Table 2.** Effect of low molecular weight chitosan (LMWC) coating on quality attributes of postharvest *Murcott* tangor fruits after 56 days of storage at 15 °C 1,2. Reproduced from Reference [15].

	**Firmness (g)**	**Total Soluble Solids (brix)**	**Titratable Acidity (%)**	**Ascorbic Acid (mg/100 mL)**	**Water Contents (%)**
Control (water)	136 ± 5 ^d^	12.9 ± 0.5 ^b^	1.12 ± 0.03 ^b^	52.2 ± 2.3 ^c^	80.7 ± 1.2 ^e^

			LMWC		
0.05%	223 ± 5 ^c^	13.8 ± 0.1 ^a^	1.25 ± 0.04 ^a^	75.1 ± 2.9 ^a^	84.1 ± 1.9 ^c^
0.1%	248 ± 5 ^b^	13.8 ± 0.2 ^a^	1.27 ± 0.05 ^a^	75.3 ± 3.1 ^a^	86.4 ± 1.2 ^b^
0.2%	269 ± 5 ^a^	13.9 ± 0.1 ^a^	1.28 ± 0.05 ^a^	75.5 ± 3.1 ^a^	87.8 ± 2.3 ^a^

			HMWC		
0.05%	196 ± 4 ^e^	13.1 ± 0.1 ^b^	1.11 ± 0.05 ^b^	63.2 ± 2.9 ^b^	83.7 ± 2.0 ^d^
0.1%	198 ± 4 ^e^	13.1 ± 0.1 ^b^	1.12 ± 0.04 ^b^	63.5 ± 3.0 ^b^	83.8 ± 1.2 ^d^
0.2%	200 ± 5 ^e^	13.2 ± 0.1 ^b^	1.12 ± 0.05 ^b^	64.1 ± 3.0 ^b^	83.9 ± 2.0 ^d^

			TBZ		
0.1%	193 ± 5 ^e^	13.1 ± 0.1 ^b^	1.12 ± 0.05 ^b^	62.7 ± 3.1 ^b^	83.5 ± 2.2 ^d^

1 Means are averaged values of three trials. Each trial contained three replicates of 120 Murcott tangor fruits per treatment.

2 Values within a column with the same letter are not significantly different (*P* > 0.05).

**Table 3.** Effects of chitosan at different concentrations with various viscosities, alone, or in combination with *Cryptococcus laurentii* on the reduction of the blue mould rot in apple fruit wounds. Reproduced from Reference [61].

**Treatments**		**Disease Incidence (%)**	**Lesion Diameter (mm)**
Control		100 ± 0	17.71 ± 1.69

1% chitosan	12 cP	39.4 ± 2.5	5.20 ± 0.17
	20 cP	45.9 ± 2.4	5.67 ± 0.29
	100 cP	51.1 ± 2.0	6.97 ± 0.35
	130 cP	55.9 ± 1.5	6.87 ± 0.35

0.1% chitosan	12 cP	87.5 ± 3.9	12.1 ± 0.32
	20 cP	100 ± 0	13.9 ± 0.31
	100 cP	100 ± 0	15.3 ± 0.60
	130 cP	100 ± 0	16.3 ± 0.63

*C. laurentii*		48.4 ± 2.6	6.67 ± 0.48

*C. laurentii +* 1% chitosan	12 cP	30.0 ± 1.3	5.52 ± 0.42
	20 cP	33.8 ± 2.7	6.37 ± 0.38
	100 cP	44.3 ± 3.2	6.93 ± 0.35
	130 cP	46.0 ± 3.4	7.47 ± 0.50

*C. laurentii +* 0.1% chitosan	12 cP	14.0 ± 1.2	2.37 ± 0.22

	20 cP	19.3 ± 2.7	3.44 ± 0.50
	100 cP	33.5 ± 2.2	6.07 ± 0.58
	130 cP	36.7 ± 1.9	6.25 ± 0.35

*C. laurentii +* 0.01% chitosan	12 cP	45.0 ± 2.7	7.7 ± 0.51
	20 cP	42.5 ± 3.7	7.46 ± 0.38
	100 cP	52.5 ± 3.1	9.46 ± 0.43
	130 cP	50.2 ± 3.3	8.67 ± 0.29

Data are means ± standard deviations of four replicates.
